# Effect of Phosphate-Based Inhibitor on Corrosion Kinetics and Mechanism for Formation of Passive Film onto the Steel Rebar in Chloride-Containing Pore Solution

**DOI:** 10.3390/ma13163642

**Published:** 2020-08-17

**Authors:** Soumen Mandal, Jitendra Kumar Singh, Dong-Eun Lee, Taejoon Park

**Affiliations:** 1 Intelligent Construction Automation Center, Kyungpook National University, 80, Daehak-ro, Buk-gu, Daegu 41566, Korea; sou.chm@gmail.com; 2 Innovative Durable Building and Infrastructure Research Center, Department of Architectural Engineering, Hanyang University, 1271 Sa3-dong, Sangrok-gu, Ansan 15588, Korea; jk200386@hanyang.ac.kr; 3 School of Architecture, Civil, Environment, and Energy, Kyungpook National University, 1370, Sangyegk-Dong, Buk-Gu, Daegu 702-701, Korea; 4 Department of Robotics Engineering, Hanyang University, 55 Hanyangdaehak-ro, Ansan, Gyeonggi-do 15588, Korea

**Keywords:** steel, concrete pore solution, corrosion, inhibitor, electrochemical impedance spectroscopy, Raman spectroscopy

## Abstract

In the present study, different contents, i.e., 1–3% of 0.5 M ammonium phosphate mono basic (APMB), were used as corrosion inhibitor to reduce the corrosion of steel rebar. Electrochemical impedance spectroscopy (EIS) results showed that up to 24 h of exposure, polarization resistance (*R_p_*) and passive/oxide film resistance (*R_o_*) gradually decreased in simulated concrete pore (SCP) + 3.5 wt.% NaCl solution owing to the reduction in pH of the solution. The steel rebar exposed in 2% inhibitor containing SCP + 3.5 wt.% NaCl solution exhibited 90% inhibition efficiency after 1 h of exposure. X-ray photoelectron spectroscopy (XPS) and Raman spectroscopy confirmed the formation of thermodynamically very stable and sparingly soluble goethite (α-FeOOH), maghemite (γ-Fe_2_O_3_), and iron phosphate (FePO_4_) as passive/oxide film onto the steel rebar surface exposed to 2% inhibitor containing SCP + 3.5 wt.% NaCl solution.

## 1. Introduction

The steel rebar is prone to corrosion in chloride-contaminated concrete environment. Chloride (Cl^−^) ions break down the passive film formed onto the steel rebar surface and cause pitting corrosion. Thus, different measures are being taken, using different protective methods to protect the steel rebar embedded in concrete from corrosion. Some of them are cathodic protection, polymeric coatings, use of inhibitors, admixtures, etc. [1,2,3,4]. However, the corrosion inhibitors have gained significant popularity owing to their easy availability and application as well as being used in a lower amount. In recent years, the use of different inhibitors is a very emerging and successful method to mitigate the corrosion of steel rebar inside the concrete [5,6]. Inhibitors delay the initiation as well as inhibit the propagation of corrosion. Three major types of inhibitors, viz., organic, inorganic, and mixed types, are being used in the concrete to reduce or delay the corrosion of the steel rebar.

Mainly, amino-based corrosion inhibitors are prevalent as organic inhibitors and commonly applied in the concrete as well as chloride containing concrete pore solution [5,7,8,9]. It was reported by Ryu et al. [7] that N, N′-Dimethyl ethanolamine inhibitor adsorbed onto the steel rebar surface and significantly reduced the corrosion initiation when exposed to NaCl-contaminated saturated calcium hydroxide solution. Alkanolamine- and amino-based inhibitors have migratory properties and can be diffused from higher to lower concentration to mitigate the corrosion of rusted steel surface. These migratory corrosion inhibitors are migrated from concrete to steel rebars surface and adsorbed by the formation of covalent bonds [4,6,10].

A significant amount of research has been carried out on the inorganic corrosion inhibitors, mostly nitrite-based, since a long time ago [4,11,12,13,14,15,16]. Calcium nitrite is an excellent inhibitor to reduce the corrosion of steel rebar in chloride-contaminated concrete environment [2,17,18,19,20]. This inhibitor works as anodic inhibitor and needs to be applied in significant amount, otherwise it causes adverse effects on the steel and concrete [21,22]. Therefore, it is very crucial to fix the amount at a suitable concentration before its application in the concrete. Lithium nitrite (LiNO_2_), i.e., nitrite-based inhibitor, has shown around 64% inhibition efficiency in reduction of steel rebar corrosion when exposed to NaCl-contaminated calcium hydroxide solution [4]. The nitrite-based inhibitors reduce the strength of the concrete by forming cracks and are also toxic in nature; therefore, it has been restricted for the use [17,23].

Due to the ecological reason, it is required to replace the nitrite-based inhibitors with eco-friendly and phosphate-based corrosion inhibitors, which are economical and less toxic. Such types of inhibitors have gained significant attention for the researchers to reduce the corrosion of steel rebar. *Bambusa arundinacea* has been used as sustainable corrosion inhibitor with nitrite and amine to study the corrosion mechanism of steel rebar in concrete environment [24]. Phosphate is one of the green inhibitors that has been used to reduce the corrosion of metals and alloys [25]. Phosphates have versatile applications in surface treatments and conversion coatings owing to nonconducting nature, which improves the corrosion resistance of the metals and alloys as well as application is also easy [26]. However, it was also reported by Jeong et al. [27] and Lee et al. [28] that phosphate can be used as a pore-sealing agent to reduce the porosity of Al coating deposited by arc thermal spray process and improve the corrosion resistance properties at a longer duration of exposure.

Phosphate-based inhibitors can be considered as an alternative to avoid the use of nitrite-based inhibitor. Generally, phosphate-based inhibitors work as migratory inhibitor [29]. Lee et al. [30] have used sodium hexameta phosphate with sodium benzoate and benzotriazole as corrosion inhibitor in SCP solution contaminated with 3.5% NaCl and they have found that as the amount of inhibitor increased, the efficiency was gradually increased up to 96% in the presence of 5% inhibitor, owing to the adsorption of inhibitor onto the steel surface. However, phosphate ions delay the onset of localized corrosion in simulated pore solution [31,32]. Aluminum triphosphate effectively hindered the pitting corrosion of the steel rebar in carbonated concrete pore solution attributed to the formation of protective passive film [33]. Sodium biphosphate was reported to reduce the pitting corrosion in chloride-contaminated carbonate concrete where 99% efficiency was observed over one month of exposure, attributed to the formation of iron phosphate onto the steel rebar surface [34]. The phosphate ions replaced the Cl^−^ ions and adsorbed onto the steel rebar surface, therefore, reduction in corrosion was observed in chloride-contaminated concrete environment [35]. Some authors have mentioned that phosphate ions may act as anodic and cathodic as well as mixed inhibitor [36,37]. Na_3_PO_4_ was reported to form a phosphate-based film that resulted in reduction of the steel rebar corrosion in concrete pore solution (pH 12.5) contaminated with chloride ions [38,39].

Ammonium phosphate is one of the phosphate-based compounds, eco-friendly, economical, and possesses versatile applications in agriculture, steel industries, and coating sectors. Lee et al. [40] have used ammonium phosphate as pore-sealing agent in Al coating applied by arc thermal spray coating process which improved the corrosion resistance by 10 times in simulated sea water. Addition of 0.1 M Ca(NO_3_)_2_ with ammonium phosphate has synergistic effect in reduction of Al coating [41].

Phosphate-based inhibitors need more research to understand the accurate mechanisms for the mitigation of steel rebar corrosion and kinetics in concrete environment. From the literature search, it was found that there were no studies carried out in the past by researchers about the effect of ammonium phosphate monobasic (NH_4_H_2_PO_4_: APMB) as inhibitor on kinetics and mechanism for corrosion mitigation of steel rebar exposed in chloride-contaminated concrete pore solution. Therefore, it was our prudent thought to study the effectiveness of APMB as inhibitor in corrosion mitigation of the steel rebar exposed in 3.5% NaCl-contaminated SCP solution. This study focused on the corrosion kinetics and mechanism of APMB inhibitor using open circuit potential, EIS, and potentiodynamic polarization with exposure periods as well as formation and characteristics of passive film onto the steel rebar surfaces by scanning electron microscopy (SEM), Raman spectroscopy, and XPS after 120 h of exposure in SCP + 3.5 wt.% NaCl solution.

## 2. Materials and Methods

### 2.1. Materials

Analytical-grade ammonium phosphate monobasic (NH_4_H_2_PO_4_: APMB) was selected as inhibitor. Firstly, 0.5 M APMB solution was prepared by dissolving 11.5 g of NH_4_H_2_PO_4_ salt in 200 mL distilled water using an automatic magnetic stirrer (MS300HS, MTOPS, Seoul, Korea) at 800 rpm and 20 (±1) °C. The pH of 0.5 M APMB solution was found to be 4.1 at 20 (±1) °C. Secondly, three different amounts, i.e., 1, 2, and 3% of 0.5 M APMB solution, were chosen and then the pH was maintained up to 9 by adding 1 M NaOH solution dropwise for each amount. The pH of the solutions was measured using digital glass pH meter (TOADKK, HM41, Takadanobaba, Shinjuku-ku, Tokyo, Japan).

The electrolytic solution, i.e., SCP solution to be used for electrochemical experiments, was prepared by dissolving analytical grade of 8.33 g/L NaOH, 3.36 g/L KOH, and 2 g/L CaO in distilled water using magnetic stirrer for 24 h at 20 (±1) °C [42,43]. Thereafter, the SCP solution was filtered using 5C number (110 mm) Wattman paper to filter out the insoluble CaO and then 3.5 wt.% NaCl was added into the filtered solution. This is called SCP + 3.5 wt.% NaCl solution. The pH of this solution was 12.65 at 20 (±1) °C.

Three different amounts, i.e., 1, 2, and 3 v/v% inhibitor (pH = 9 as discussed above), were added into the SCP + 3.5 wt.% NaCl solution for electrochemical experiments. After addition of 1, 2. and 3% inhibitor in SCP + 3.5 wt.% NaCl, the pH of resulting solutions was measured and found to be 12.65, 12.67. and 12.68 at 20 (±1) °C, respectively.

A 16 mm diameter of steel rebar was cut from 1000 mm length into 10 mm thickness to investigate the effect of corrosion inhibitor in SCP + 3.5 wt.% NaCl solution. The elements and their amounts (wt.%) in the steel rebar samples were C = 0.235, Si = 0.250, P = 0.014, S = 0.006, Ni = 0.028, Mn = 0.90, Cr = 0.037, Mo = 0.009, Cu = 0.018, Sn = 0.002, and Fe the remainder. Before starting the electrochemical studies, the steel rebars were pickled with 10 v/v% hydrochloric acid solutions to remove the black oxide layers from the surface, washed with distilled water, rinsed thoroughly with ethanol, and dried. Furthermore, the steel rebars were mechanically polished on emery papers (220 to 2400 grit size), followed by cloth polishing using alumina (0.5-μm particle size) slurry to achieve the defect free mirror finished surface. Finally, the polished samples were degreased with acetone and used for the electrochemical studies.

### 2.2. Electrochemical Studies

The electrochemical experiments were performed by taking 0.78 cm^2^ exposure area of steel rebars at prolonged duration, i.e., up to 120 h. It is very important to stabilize the open circuit potential (OCP) of steel rebar prior to starting the electrochemical studies. Thus, the steel rebars were exposed in SCP + 3.5 wt.% NaCl (bare) as well as different amounts, i.e., 1, 2, and 3%, APMB inhibitor added in SCP + 3.5 wt.% NaCl solution for 30 min, as shown in Table 1. All experiments were performed at 20 °C in triplicate sets of steel rebar samples and averages were recorded as results. The electrochemical experiments were carried out by three electrode systems where steel rebar sample acted as working electrode (WE), platinum wire as counter electrode (CE), and Hg/Hg_2_Cl_2_ (saturated calomel electrode: SCE) as reference electrode (RE). The WE and the RE were fixed at the minimum distance to minimize the solution resistance. The exposure area of CE was greater than WE to provide the conducting path and flow of electrical current inside the solution. The EIS experiments were conducted at OCP from 100 kHz to 0.01 Hz with 10 mV sinusoidal voltage. Potentiodynamic polarization experiments were performed using a potentiostat (VersaSTAT, Princeton applied Research, Oak Ridge, TN, USA) at a 1 mV/s scan rate ranging from −0.4 V to +0.8 V vs. SCE. The data obtained with the potentiostat were analyzed by fitting the experimental data in constant phase element (CPE) model using Metrohm Autolab Nova 1.10 software.

### 2.3. Characterization

The steel rebar samples were kept for 120 h exposure in bare and 2% APMB-added solutions to form proper passive film and, thereafter, morphologies were examined by SEM (MIRA3, TESCAN, Brno, Czech Republic) equipped with energy-dispersive X-ray spectroscopy (EDS) at 15 kV.

The nature of passive film formed onto the steel rebar surfaces after 120 h of exposure in bare and 2% APMB inhibitor-added solutions were characterized by Raman spectroscopy (Horiba, LabRAM HR, Villeneuve d’Ascq, France) using He-Cd diode laser beam with a 325-nm wavelength. During the collection of Raman spectra, the laser power (1 mW) of the Raman instrument was kept low as much as possible for 10 s. Samples were scanned from 200 cm^−1^ to 600 cm^−1^.

XPS (Scienta Omicron R3000, Taunusstein, Germany) was performed with Al *K*_α_ (1486.6 eV) X-ray as source of radiation to understand the chemical states of the elements present in the passive film formed onto the steel rebar surface after 120 h of exposure in bare and 2% APMB inhibitor-added solutions. The collected XPS spectra were calibrated with C 1s peak of adventitious carbon (284.6 eV binding energy). The high-resolution peaks for individual elements were deconvoluted and fitted with Gaussian/Lorentzian function using CASA XPS software (Casa Software Ltd., Teignmouth, UK) after the background correction using Shirley method.

## 3. Results

### 3.1. Electrochemical Studies

#### 3.1.1. OCP Measurements with Exposure Periods

**Figure 1:**
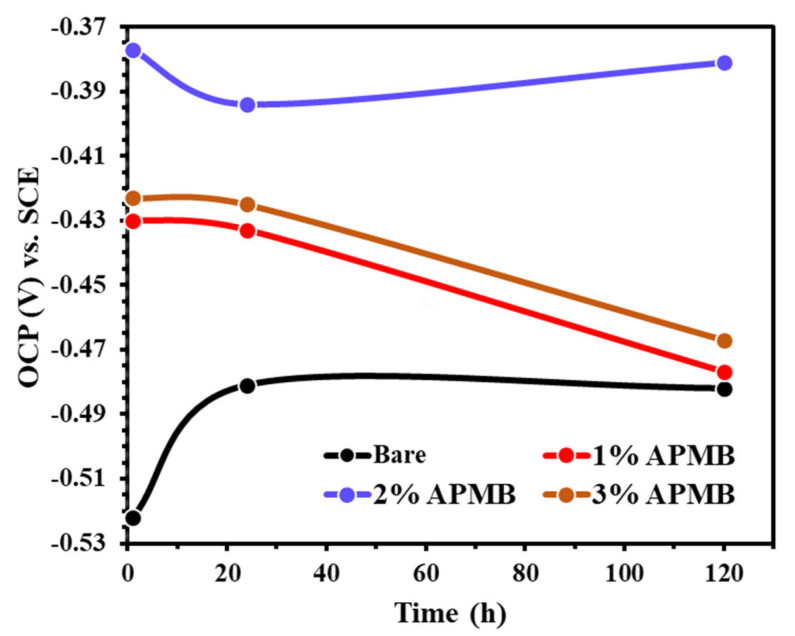
OCP plot of steel rebars exposed in SCP + 3.5 wt.% NaCl (bare) solution and inhibitor-added SCP + 3.5% NaCl solutions.

The OCP of steel rebar samples exposed in SCP + 3.5 wt.% NaCl (bare) as well as 1, 2, and 3% inhibitor-added SCP + 3.5 wt.% NaCl solutions with different exposure periods are shown in Figure 1. It can be seen from Figure 1 that the steel rebar samples exposed to the inhibitor-containing solutions show nobler OCP compared to without inhibitor- (bare) containing solution owing to the formation of protective passive film, which blocks the active sites of steel rebar from the attack of Cl^−^ ions. Earlier reports by Lee et al. [30] and Bastidas et al. [44] well corroborate our findings where the phosphate-based corrosion inhibitors reduced the steel corrosion. In the present study, the steel rebar sample exposed to the bare solution showed active OCP, owing to the presence of 3.5 wt.% NaCl, which induces the localized corrosion, [45] whereas, with the steel rebars exposed to the various amounts of APMB inhibitor-added solutions, the OCP shifted towards nobler direction, owing to the formation of passive film [46]. The nature of phosphate-based inhibitor can be decided on the basis of concentration whether it acts as cathodic or anodic [39]. However, the steel rebar sample exposed to the bare solution exhibited more active OCP compared to inhibitor-added SCP + 3.5 wt.% NaCl solutions with exposure periods. In this case, Cl^−^ ions locally attacked and induced the pitting corrosion, resulting in formation of oxide film. The steel rebar exposed to 2% inhibitor-added SCP + 3.5 wt.% NaCl solution showed noble OCP among all samples, but once the inhibitor concentration was lower or higher than 2%, the OCP shifted towards active direction. The 1% inhibitor was not an optimum amount, where Cl^−^ ions can hinder the formation of passive film. Therefore, active OCP was observed. On the other hand, the steel rebars exposed in 3% inhibitor-added solution showed active OCP with time compared to the sample 2% inhibitor, owing to the local acidification by anodic dissolution of iron and hydrolysis of ferric chloride [46], which increased the conductivity of passive film. Moreover, the steel rebar exposed to 3% inhibitor-added solution showed nobler OCP throughout the exposure periods compared to bare and 1% inhibitor. The steel rebar samples exposed to 1% and 3% inhibitor-containing solutions exhibited gradual increment in OCP toward active direction from 1 h to 120 h. However, the steel rebar exposed in 2% inhibitor-containing solution exhibited active OCP up to 24 h, but once the exposure periods were increased, it shifted toward nobler direction, owing to the formation of protective passive film. The OCP of the steel rebar exposed to 2% inhibitor-containing solution was nobler compared to others in all exposure periods. This result indicated that 2% is the optimum amount of 0.5 M APMB inhibitor, which led to forming phosphate layer for proper protection of steel sample exposed in SCP + 3.5 wt.% NaCl solution [47].

#### 3.1.2. EIS Studies with Exposure Periods

**Figure 2:**
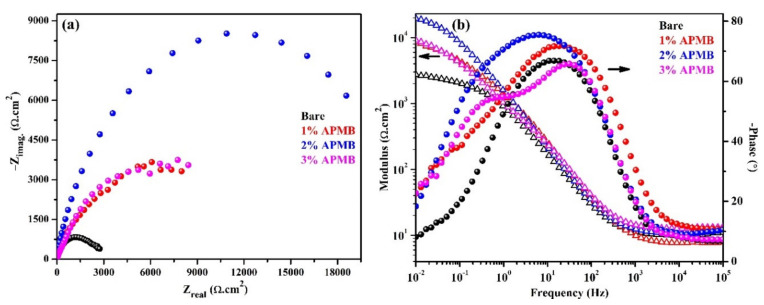
EIS (**a**) complex plane impedance and (**b**) modulus-frequency and phase-frequency Bode plots of steel rebars after 1 h of exposure in SCP + 3.5 wt.% NaCl (bare) solution and inhibitor-added SCP + 3.5 wt.% NaCl solutions.

The EIS analysis is an important technique to understand the underlying corrosion mechanism. Thus, in the present study, EIS analysis was carried out to understand the effect of inhibitor in the corrosion protection of steel rebar. The complex plane impedance and Bode plots of steel rebars after 1 h of exposure in bare and inhibitor-added SCP + 3.5 wt.% NaCl solutions are shown in Figure 2a,b, respectively. From complex plane impedance plots (Figure 2a), it can be seen that the steel rebar samples exposed to inhibitor-containing solutions exhibited higher magnitude in complex plane impedance (Nyquist) plots compared to bare solution. However, the steel rebar sample exposed to 2% inhibitor-added SCP + 3.5 wt.% NaCl solution exhibited highest in the magnitude of real and imaginary component of complex plane impedance compared to other samples, attributed to the formation of passive film onto the surface [10]. The Cl^−^ ions attacked onto the steel rebar surface, which led to the corrosion phenomena and, therefore, the magnitude in complex plane impedance of the sample exposed to bare solution was the lowest among all samples. There is the possibility that, at longer duration of exposure, the steel rebars in SCP + 3.5 wt.% NaCl solution (bare) induced the corrosion, owing to the high content of Cl^−^ ions in the solution. This result corroborated our OCP observation (Figure 1), where it was shifted toward active direction after 1 h of exposure. The complex plane impedance of the steel rebars exposed to 1% and 3% inhibitor-added solution show almost identically in real and imaginary component magnitude.

The modulus-frequency and phase-frequency Bode plots of steel rebars after 1 h of exposure to the bare and inhibitor-added solutions are shown in Figure 2b. The lowest impedance value is shown by the steel rebar sample exposed to SCP + 3.5 wt.% NaCl (bare) solution, while the highest is shown by the sample exposed to 2% inhibitor-added solution. Therefore, this result suggests that neither 1% nor 3% inhibitor additions were providing significant protection to the steel rebar from corrosion exposed in SPC + 3.5 wt.% NaCl solution. The lowest impedance value was observed for the steel rebars exposed in bare solution, owing to the initiation of corrosion where Cl^−^ ions locally attacked on the active sites of the steel rebar. The steel rebar exposed in 2% inhibitor-added solution formed the passive oxide film and covered all over the surface, which stifled the attack of Cl^−^ ions, whereas 1% inhibitor was not substantial to form uniform film. Thus, the lower impedance was observed for the steel rebar exposed in 1% inhibitor-added SCP + 3.5 wt.% NaCl solution. On the other hand, for the steel rebar exposed in 3% inhibitor-added SCP + 3.5 wt.% NaCl solution, the localized pH at the interface of the steel rebar/solution was reduced thereby, and lower impedance was observed at 0.01 Hz compared to 2% inhibitor after 1 h of exposure.

Phase-frequency Bode plots of steel rebars after 1 h of exposure to the bare and inhibitor-added solutions exhibited two times constant (Figure 2b), which revealed that there would be one electrical equivalent circuits (EEC) fitted. The first time constant in EEC from higher to middle frequency was attributed to the steel/solution interface while another from middle to lower frequency was attributed to the passive film/solution interface. The phase angle maxima of the steel rebar exposed to inhibitor-added solutions were shifted toward higher angle in middle as well as lower studied frequencies, revealing the formation of passive films, whereas the samples exposed without inhibitor (bare) showed shifting of maxima in lower angle (Figure 2b). The phase angle maxima of the steel rebar sample exposed to bare solution showed at −67° on 14 Hz and −13° on 0.03 Hz. The shifting of phase angle maxima at −67° suggests that passive film is inhomogeneous, defective, and porous, which develop corrosion products at longer duration of exposure. However, the steel rebar exposed to 1% inhibitor-containing solution exhibited two phase angle maxima at −72° on 18 Hz and −38° on 0.08 Hz. The shifting of phase angle maxima in middle to lower studied frequencies revealed the formation and strengthening of passive film. Moreover, the steel rebars exposed in 2% inhibitor-added solution exhibited the phase angle maxima at −75° from 30 Hz to 0.3 Hz. The broadening and asymmetrical peak for this sample suggested that passive film was uniform, homogenous, and protective. Thus, the impedance was found to be highest among all samples after 1 h of exposure [4,5,11]. However, at the lowest studied frequency, i.e., 0.01 Hz, the phase angle maxima were shifted at −18°, which meant that steel surface was being protected by passive film. The steel rebar exposed in 3% inhibitor-containing solution showed two maxima at −66° and −54° on 32 Hz and 0.43 Hz, respectively. The shifting of phase angle maxima on 32 Hz was attributed to the defective passive film, which was caused by acidification of solution and led to form porous oxide film. This porous oxide film acted as a barrier for penetration of aggressive ions from solution to steel rebar surface. However, due to the acidification, the corrosion product started to dissolve. Therefore, it was utmost required to continue the EIS at longer duration. In the subsequent paragraphs, the corrosion kinetics and mechanism of corrosion phenomena after 24 h and 120 h of exposure will be discussed.

**Figure 3:**
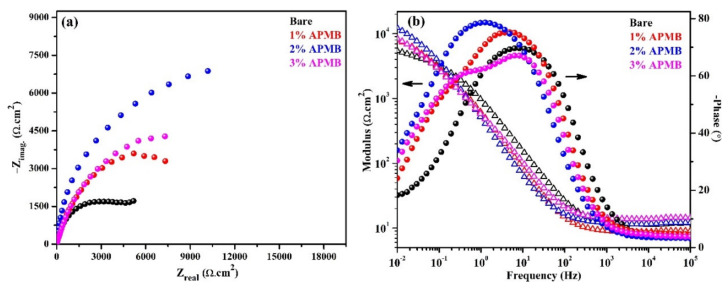
EIS (**a**) complex plane impedance and (**b**) modulus-frequency and phase-frequency Bode plots of steel rebars after 24 h of exposure in SCP + 3.5 wt.% NaCl (bare) solution and inhibitor-added SCP + 3.5 wt.% NaCl solutions.

The magnitude of complex plane impedance plots of the steel rebar samples exposed in inhibitor-added SCP + 3.5 wt.% NaCl solution after 24 h of exposure was more decreased than 1 h exposure, whereas, in the case of bare solution, it was increased (Figure 3a). This result suggested that, owing to the initiation of the corrosion process in bare solution at steel rebar/solution interface after 1 h of exposure, corrosion/oxide film started to form, which stifled the ingress of Cl^−^ ions and, therefore, an increase in magnitude was observed (Figure 3a). The steel rebar samples exposed to the inhibitor-containing SCP + 3.5 wt.% NaCl solutions exhibited higher in the magnitude of complex plane impedance compared to bare solution, even after 24 h of exposure. It meant the passive film formed onto the steel rebar surface but, due to the acidification of solution by APMB, it was not stable. The steel rebars exposed in 2% and 3% inhibitor-containing solutions showed higher in the magnitude of −Z_imag_ after 24 h of exposure compared to 1 h, owing to the capacitive properties of the passive film [11]. This result suggested that corrosion products or oxide film started to form. The magnitude of complex plane impedance plots of steel rebars exposed in 2% inhibitor solution was the highest, followed by 3%, 1% inhibitor-containing solution, and bare solution.

The modulus-frequency Bode plots of the steel rebar samples after 24 h of exposure are shown in Figure 3b. From this Figure, it is depicted that the impedance value at 0.01 Hz decreased in the case of inhibitor-containing samples compared 1 h exposure while bare solution sample slightly increased, owing to the formation of corrosion products. Due to the thickening of oxide film, impedance was increased. However, it is not necessary that corrosion products are stable. Thus, it was required to study the actual nature of corrosion products at longer duration of exposure. In the case of inhibitor, there would be two types of effects dominating on steel rebar/solution interface for corrosion. One may be due to the attack of Cl^−^ ions and another for localized acidification of solution by H_3_PO_4_ released from APMB. Cl^−^ (from NaCl) and H^+^ (H_3_PO_4_) ions both participate in corrosion reaction. If passive/oxide film is weak, then initiation and propagation of corrosion reaction would occur. There is a possibility that if phosphate ion is significant to transform the weak and loose oxides, i.e., Fe(OH)_2_ into protective and stable oxide film, then corrosion products would reduce the corrosion reaction. The transformation of Fe(OH)_2_ requires some time to form the protective oxide film. Thus, it was required to extend the exposure periods beyond 24 h. The corrosion mechanism after 120 h of exposure is discussed in the subsequent paragraphs.

The phase-frequency Bode plots of samples after 24 h of exposure are depicted in Figure 3b. From this figure, it can be seen that the phase angle maxima are found to be at −69° on 6 Hz, with the steel rebar exposed to bare solution. This phase angle maxima shift suggested that corrosion products are thick and porous (due to capacitance) rather than compact and uniform. Due to the thick corrosion products, the total impedance was found to be slightly higher after 24 h of exposure compared to 1 h. This finding suggested that Cl^−^ ions initially attacked and initiated the localized corrosion and resulted in thick corrosion product onto the steel rebar/solution interface. There was another phase angle maxima at −18° on 0.01 Hz due, to the steel substrate. This value was identical to that obtained after 1 h of exposure, which indicated that steel was not getting any other protection rather than corrosion products. For the steel rebar samples exposed to 1% inhibitor-added solution, its phase angle maxima shifted at −75° on 6 Hz and −24° on 0.1 Hz after 24 h of exposure, whereas after 1 h of exposure, it was found at −72° on 18 Hz and −38° on 0.08 Hz. The shifting of phase angle maxima at higher angle in low frequency, i.e., 0.08 Hz, revealed the corrosion protection of the steel rebar. On the other hand, the steel rebar sample exposed in 2% inhibitor-added solution exhibited two time maxima at −79° and −34° on 1 Hz and 0.01 Hz, respectively. It is reported that if phase angle maxima shifted around −80°, then the passive film is homogenous and protective [30]. However, the steel rebar exposed in 3% inhibitor-containing solution showed two distinct phase angle maxima at −67° and −59° on 6 Hz and 0.25 Hz, respectively.

**Figure 4:**
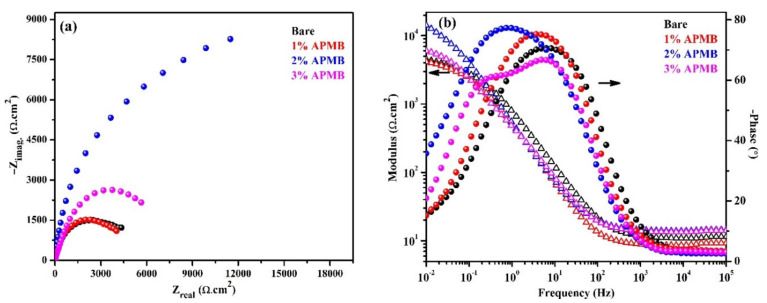
EIS (**a**) complex plane impedance and (**b**) modulus-frequency and phase-frequency Bode plots of steel rebars after 120 h of exposure in SCP + 3.5 wt.% NaCl (bare) solution and inhibitor-added SCP + 3.5 wt.% NaCl solutions.

EIS experiments were carried out up to 120 h of exposure, and the results are presented in Figure 4. The complex plane impedance plots are depicted in Figure 4a after 120 h of exposure. The steel rebar sample exposed in bare solution exhibited reduced magnitude in complex plane impedance, owing to the porous oxide film. As discussed earlier, after 24 h of exposure, corrosion products were porous, which are good reservoirs of moisture, oxygen, and aggressive ions that influence the corrosion. Thus, reduced magnitude was observed after 120 h of exposure compared to 24 h. Besides, the steel rebars exposed to 1% and 3% inhibitor-added solutions were also exhibiting reduction in the magnitude of complex plane impedance, owing to the propagation of corrosion reaction. In the case of steel rebar sample exposed to 1% inhibitor-added solution, the magnitude of impedance was lower than bare, owing to the synergistic effect of Cl^−^ (NaCl) and H^+^ (H_3_PO_4_) ions. Cl^−^ ions induced the corrosion by the localized attack, whereas H^+^ acidified the solution. In this case, the phosphate ions were not significant to transform the unstable corrosion products into protective oxide film and simultaneously the Cl^−^ ions induce the deterioration of formed passive film. Thus, lower in the magnitude was observed (Figure 4a). The 2% inhibitor might have been the optimum, which led to forming the iron phosphate, and Cl^−^ ions were not able to break the film. Thus, the highest magnitude was observed. It can be seen that the complex plane impedance magnitude was higher compared to 24 h of exposure, attributed to the formation of very protective passive/oxide film. On the other hand, the steel rebar sample exposed in 3% inhibitor-added solution after 120 h showing lower in the magnitude compared to 24 h was attributed to the reduction in localized pH, which acidifies the SCP + 3.5 wt.% NaCl solution. In this case, higher amount of H_3_PO_4_ would form, which would reduce the pH significantly. Thus, at longer duration of exposure, complex plane impedance magnitude was reduced. Even after 120 h of exposure, the magnitude of the sample in 3% inhibitor-added solution was higher compared to 1% and bare, but lower than 2% inhibitor-added solution.

The modulus-frequency and phase-frequency Bode plots of the samples after 120 h of exposure are shown in Figure 4b. From this figure, it is depicted that the steel rebar samples exposed to 2% inhibitor-added solution exhibited an increase in total impedance, attributed to the formation of protective passive/oxide film compared to 24 h of exposure, whereas, the steel rebar exposed in bare, 1%, and 3% inhibitor-added solution showed decrease in its value at 0.01 Hz, owing to the propagation of corrosion reaction. The decrease in impedance values of these samples was attributed to the different reasons. In the case of steel rebar sample in bare solution, Cl^−^ ion induced the corrosion reaction, owing to the porous oxide film where moisture and oxygen were significant. In the case of 1% inhibitor-added solution, Cl^−^ and H^+^ ions both were participating in corrosion reaction. Thus, lower impedance value was observed (Figure 4b). However, for 3% inhibitor-added solution, there might be competition between Cl^−^, H^+,^ and phosphate ions. Cl^−^ and H^+^ ions induced the corrosion reaction but at the same time, phosphate ion reacted with corrosion products, i.e., Fe(OH)_2,_ and transformed into protective corrosion products/oxide film, which diminished the effect of Cl^−^ and H^+^ ions. Thus, higher impedance was observed compared to bare and 1% inhibitor-added solution after 120 h of exposure. Moreover, the lower impedance, compared to 24 h, was attributed to the effect of Cl^−^ and H^+^ ions.

The phase-frequency Bode plots after 120 h of exposure corroborate with complex plane impedance and modulus-frequency Bode where steel rebar sample exposed in bare solution exhibited a sharp peak at −70° on 6 Hz, whereas at the lowest studied frequency, i.e., 0.01, Hz was found to be at −15°. At lowest frequency, phase angle was decreased compared to 24 h (−18° at 0.01 Hz). The presence of a sharp, asymmetric peak at middle frequency and lower in phase angle maxima at 0.01 Hz, implied the deterioration of steel rebar after 120 h of exposure. The steel rebar sample exposed in 1% inhibitor-added solution shows phase angle maxima shift at −75° on 3 Hz, which revealed the deterioration of steel rebar surface, but the film was homogenous. Due to the deterioration of steel surface, rather than formation of stable corrosion products, the impedance was decreased. Broadening in phase angle maxima of the sample exposed in 2% inhibitor-added solution at −77° from 2 Hz to 0.2 Hz and −36° on 0.01 Hz revealed the formation of homogenous, uniform, and protective oxide/passive film, which stifled the attack of Cl^−^ ions. Thus, the highest impedance was observed after 120 h of exposure. However, 3% inhibitor exhibited phase angle maxima at −67° and −60° on 11 Hz and 0.1 Hz with broadening, respectively, which revealed that the passive film was uniform and protective compared to the samples exposed in bare and 1% inhibitor-added solution. Moreover, it can be seen at 0.01 Hz that phase angle was shifted towards lower angle, i.e., −21° compared to 24 h i.e., −37°. This result suggested that 3% inhibitor-containing solution induced the corrosion of steel rebar surface, due to the acidification where steel rebar starts to deteriorate. Thus, total impedance decreased compared to 24 h of exposure (Figure 4b).

**Figure 5:**
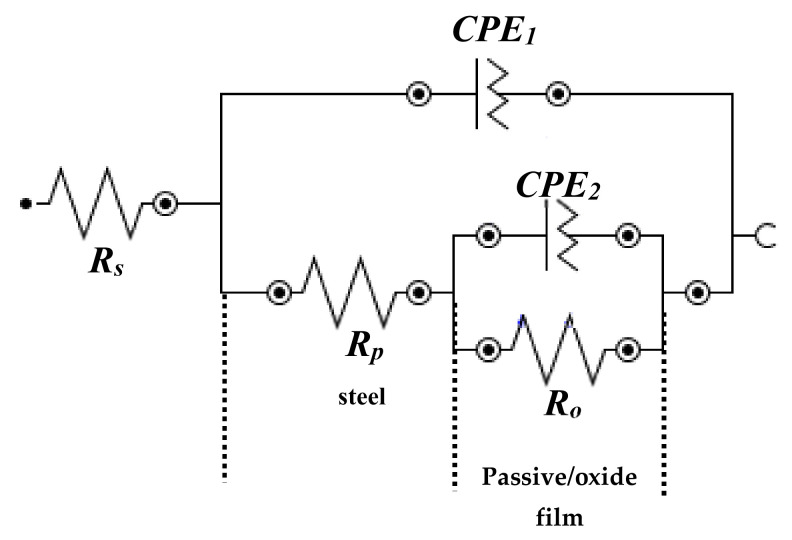
EEC of steel rebar samples exposed in SCP + 3.5 wt.% NaCl solution with and without inhibitor.

The EIS results indicated that all samples exhibited two time constants, from high to middle and middle to lower studied frequencies. Therefore, all EIS plots could be fitted into one EEC [31,32,46,48]. The first time constant was associated with corrosion reaction at steel/solution interface in high to middle frequencies, whereas second at passive or oxide film/solution interface in middle to low frequencies (Figure 5). The first time constant in EEC was associated with solution resistance (*R_s_*), constant phase element (CPE1), and *R_p_*. Due to the corrosion and heterogeneity of the steel surface, instead of pure capacitance, CPE participated. The CPE1 and *R_p_* were associated parallel to each other. The second time constant in EEC, CPE2, and *R_o_* or charge transfer were parallel to each other.

Based on EIS fitting, CPE coefficient (*Q_eff_*) can be calculated from imaginary impedance (*Z_j_*) when CPE exponent (*n*) ≠ 1, by Equation (1) [49]:

(1) $$Q_{eff}=\sin\left(\frac{n\pi }{2}\right)\frac{-1}{Z_{j}(f){\left(2\pi f\right)}^{n}}$$

where *f* is frequency. But, once *n* becomes 1, then *Q_eff_* becomes a capacitance (*C_eff_*). Therefore, Equation (1) can be written as:

(2) $$Q_{eff}=C_{eff}=\frac{-1}{Z_{j}(f)(2\pi f)}$$

However, it is not possible for *n* to become 1, owing to the heterogeneity of the steel rebar surface during exposure in SCP + 3.5 wt.% NaCl solution, where corrosion reaction has started once exposed in solution. Therefore, the blocking characteristics by corrosion products on steel surface between the interfacial capacitance and CPE coefficient (*Q*) can be calculated by Brug’s equation [50] and others [51,52]:

(3) $$C_{eff}=Q^{1/n}R_{S}^{(1-n)/n}$$

**Figure 6:**
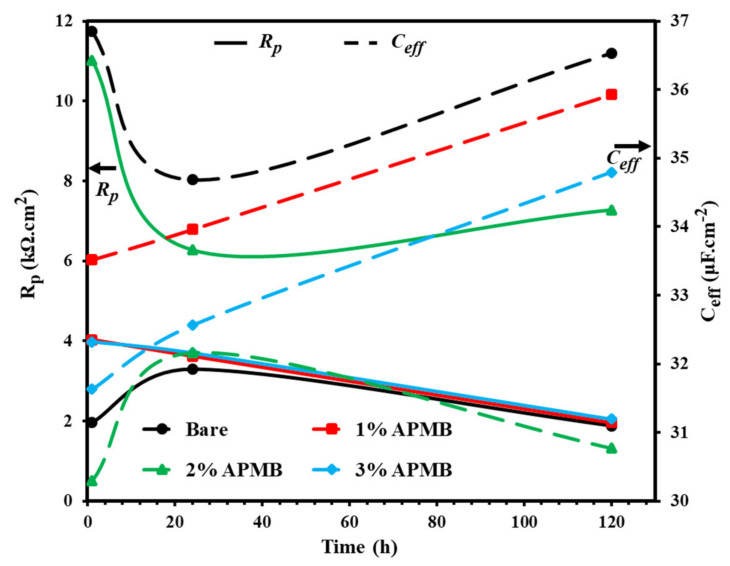
*R_p_* and *C_eff_* plots of steel rebars exposed in SCP + 3.5 wt.% NaCl solution with and without inhibitor at different exposure periods.

The electrochemical parameters obtained from EIS plots’ fitting in suitable EEC (Figure 5) with exposure periods are shown in Table 2 and Figure 6. From this table, it can be seen that *R_s_* was found in between 9.96 to 12.89 Ω·cm^2^ at different exposure periods with and without inhibitor. This result suggests that the conductivity of the solution remained constant throughout the exposure periods with and without inhibitor. The *R_p_* value of the steel rebar sample in bare (without inhibitor) solution was exhibiting the lowest, i.e., 1.98 kΩ·cm^2^, while *C_eff_* was the highest, i.e., 36.85 μF·cm^−2^, after 1 h of exposure (Figure 6) compared to inhibitor-added solutions, owing to the localized attack of Cl^−^ ion where initiation of corrosion phenomena started. At the same time, however, during initiation of corrosion phenomena, corrosion products would deposit onto the steel rebar surface and increase the *R_p_* value. After 24 h of exposure, it was found to be 3.29 kΩ·cm^2^. As the exposure periods were extended up to 120 h, the *R_p_* value of the steel rebar samples exposed to bare solution was decreased (Figure 6) compared to 24 h, owing to the formation of loose, nonprotective, and porous oxide film onto the surface, which worked as a reservoir for collecting ions, oxygen, and moisture that enhanced the corrosion reaction. The *R_p_* and *C_eff_* of the steel rebar samples exposed in inhibitor-containing solutions were higher and lower, respectively, than the sample of bare solution, in all exposure periods, owing to the formation of corrosion products and adsorption of inhibitor onto the steel rebar surface. The *R_p_* values of steel rebar samples exposed in 1 and 3% inhibitor-containing solutions were gradually decreased and *C_eff_* was increased from 1 h to 120 h (Figure 6). In 1% inhibitor, phosphate ion was not significant to form protective film and, simultaneously, Cl^−^ ions locally attacked, which induced the corrosion reaction. While in the case of 3% inhibitor, there was possibility for acidification where H^+^ ions (from H_3_PO_4_) hinder the formation of protective film and simultaneously started to deteriorate the steel rebar surface. In the case of 2% inhibitor-added solution, *R_p_* value was decreased from 11.02 kΩ·cm^2^ to 6.28 kΩ·cm^2^ for 1 h to 24 h, respectively, attributed to Cl^−^ and H^+^ ions, which induced the corrosion reaction. The formed corrosion products/oxide film up to 24 h required incubation periods to react with phosphate ions and transform into protective passive/oxide film. Once the exposure periods reached 120 h, the loosely bound oxide reacted with phosphate ions and then transformed the nonprotective oxide into protective oxide. Thus, *R_p_* was found to be highest, i.e., 7.28 kΩ·cm^2,^ and *C_eff_* lowest, i.e., 30.77 μF·cm^−2^.

From Table 2, it can be seen that *Q_1_* values of steel rebar samples up to 24 h of exposure in bare solution were decreased but remained higher than inhibitor-added solutions, owing to the increase in *R_p_* value. Once the exposure period was extended up to 120 h, *Q_1_* increased, owing to the heterogeneity of the surface, attributed to the corrosion reaction. The *n_1_* values revealed that steel surface was heterogeneous, as its value was found to be 0.76 after 120 h of exposure. *Q_1_* values of all rebar samples exposed to inhibitor-added solutions were found to be lower up to 24 h of exposure than the bare solution, which revealed that the surface was homogenous. This result correlates with the *n_1_* values where it is more than 0.8. It was reported by other researchers that, if *n* value is more than 0.8, then the passive film formed onto the steel rebar surface is homogenous [48,53].

Once the steel rebar was exposed in SCP + 3.5 wt.% NaCl solution with and without inhibitor, the corrosion reaction started at steel rebar/solution interface resulted in formation of passive/oxide film. Thus, *R_o_* or charge transfer resistance were involved in corrosion phenomena. From Table 2, it was observed that *R_o_* value of the steel rebar samples exposed in 1 and 3% inhibitor-added solution gradually decreased, attributed to the synergistic effect of Cl^−^ ions and acidification. In 1% inhibitor, once the corrosion reaction started, Cl^−^ ions locally attacked, which induced the corrosion reaction to result in the formation of corrosion products as well as, in the meantime, H^+^ ions being entrapped in the corrosion products, which acidified the SCP + 3.5 wt.% NaCl solution with exposure periods. In this case, phosphate ions were not in a significant amount to react with corrosion products and transform the nonprotective corrosion products into stable and protective. Thus, after 120 h of exposure, *R_o_* was found to be lower, i.e., 2.26 kΩ·cm^2,^ than bare, i.e., 2.66 kΩ·cm^2^ (Table 2). Moreover, *Q_2_* was higher and *n_2_* was lower than the steel rebar samples exposed to bare solution. The *n_2_* values revealed that corrosion products were heterogeneous and porous in nature. On the other hand, *n_2_* of steel rebar sample exposed in 3% inhibitor-added solution with exposure periods was decreased but remained greater than sample in bare solution and below 2% inhibitor-added solution. It was attributed to the positive effect of phosphate ions, which initially, i.e., up to 24 h of exposure to significant amount of corrosion products, would have been formed, owing to the greater amount of H^+^ ions, which influenced the corrosion reaction. Due to the acidification of solution, corrosion products started to dissolve at longer duration, i.e., 120 h of exposure. Thus, *R_o_* and *n_2_* decreased and *Q_2_* increased. This result suggests that even after 120 h of exposure, corrosion product formed on steel rebar surface in 3% inhibitor-added solution was protective compared to the sample in bare solution. The steel rebar samples exposed in 2% inhibitor-added solution showed the highest *R_o_* value in all exposure periods compared to bare, 1, and 3% inhibitor-added solutions, owing to the formation of protective, adherent, and stable corrosion products. Up to 24 h of exposure, *R_o_* was decreased but once the exposure periods were extended to 120 h, *R_o_* value increased, owing to the transformation of corrosion products. Initially, due to the Cl^−^ ions, corrosion was induced. Thus, corrosion products formed, which again reacted with an optimal amount of phosphate ions and transformed into very stable corrosion products. Therefore, *Q_2_* decreased. The *n_2_* value around 0.8 indicated that, even after 120 h of exposure, corrosion products were homogenous and protective, which enhanced the corrosion-resistance properties of steel rebar exposed in SCP + 3.5 wt.% NaCl solution.

The efficiency (%) of inhibitor can be calculated by [31]

(4) $$Efficiency\;(\%)=\left[1-\frac{R_{0\;(bare)}}{R_{0\;(with\;inhibitor)}}\right]\times 100$$

where $R_{0\;\left(bare\right)}$ and $R_{0\;\left(with\;inhibitor\right)}$ are passive/oxide film resistance or charge transfer resistance formed onto the steel rebar surface of bare (without) and with inhibitor-added solution, respectively.

The efficiency (%) of steel rebar exposed in 1, 2, and 3% inhibitor-added SCP + 3.5 wt.% NaCl solution was found to be 81.13, 90.01, and 83.40% after 1 h of exposure (Table 2), respectively. As the exposure periods were increased, the efficiency values were decreased gradually. It could be seen that the steel rebar sample exposed in 2% inhibitor-added solution maintained its efficiency greater than 60% up to 120 h of exposure. Yuhai et al. [31] studied the effect of phosphate ion on corrosion inhibition of steel rebar exposed in simulated concrete pore solution with Cl^−^ ion and they found that a maximum 51.13% inhibition efficiency can be achieved after 24 h of exposure.

#### 3.1.3. Potentiodynamic Polarization Studies after 120 h of Exposure

**Figure 7:**
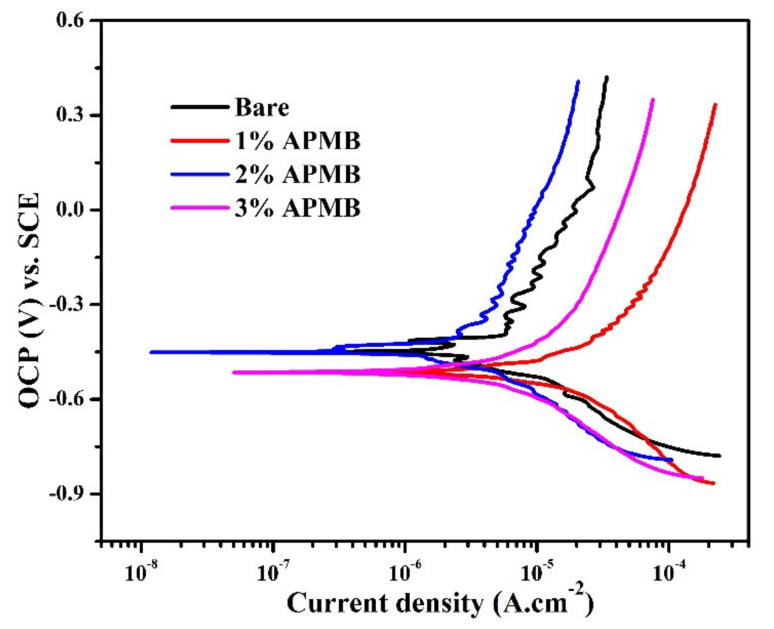
Potentiodynamic polarization curves of steel rebar samples after 120 h of exposure in SCP + 3.5 wt.% NaCl solution with and without inhibitor.

The potentiodynamic polarization plots of steel rebars exposed in SCP + 3.5 wt.% NaCl solution with and without inhibitor are shown in Figure 7. From this figure, it can be seen that cathodic current density of the steel rebar samples exposed to inhibitor-containing solutions was lower than bare, owing to the formation of oxide film. It can be seen from Figure 7 that cathodic current densities of the samples exposed in bare and 1% inhibitor-added solutions were higher compared to 2 and 3% inhibitor-added solutions, owing to the oxygen reduction reaction, which led to forming porous oxide layer. The formation of porous oxide film onto the steel rebar surface exposed to bare solution was attributed to the attack of Cl^−^ ions that remained in oxide and later caused corrosion. While the steel rebar sample was exposed to 1% inhibitor-added solution, Cl^−^ and H^+^ (from H_3_PO_4_) as well as phosphate ions were present. Cl^−^ ions remained in corrosion products and H^+^ induced the dissolution of corrosion products, while phosphate ions allowed to transform some oxides into stable ones. However, due to the presence of Cl^–^ and H^+^ ions, it would not be stable. Thus, anodic current density was highest (Figure 7). On the other hand, the steel rebar samples exposed in 2 and 3% inhibitor-added solutions showed almost identical cathodic current density but lower than bare and 1% inhibitor-added solution, owing to formation of stable and protective corrosion products. Moreover, 2 and 3% inhibitor contained a significant amount of phosphate ions, which transformed the porous oxide film into a stable one.

The anodic current density of the steel rebar samples exposed in 2% inhibitor-added solution was the lowest followed by bare, 3, and 1% inhibitor-added solutions. In the case of 2% inhibitor-added solution, the phosphate ions might have been optimal, which would significantly lead to oxidize and transform the nonprotective, loose, and porous oxide film into stable and protective. The 3% inhibitor-added solution contained the highest amount of H^+^ and phosphate ions, which initially allowed forming of the protective oxide film. However, during cathodic scanning, H^+^ ions were released in solution, which led to dissolving the oxide film. Due to the release of a higher amount of H^+^ ions in 3% inhibitor-containing solution, anodic current density was higher compared to the sample exposed in bare solution. It can be seen from Figure 7 that samples in bare and 2% inhibitor-added solutions exhibited many breakdown OCP, which indicated that during anodic scanning many times passive film was formed and broken down and, therefore, zig-zag plots were observed. It was attributed to the instantaneous formation of semi-conducting passive film and breakdown by Cl^−^ ions. There was an abrupt increment in current density after corrosion potential (*E_corr_*) for the samples exposed in 1 and 3% inhibitor-added solutions, which revealed the occurrence of pitting corrosion (Figure 7).

It can be seen from Figure 7 that *E_corr_* of the steel rebar exposed in 2% inhibitor-added and bare solutions shifted towards nobler, while the samples in 1 and 3% inhibitor-added solutions were on active direction. The *E_corr_* of the steel rebar exposed to 2% inhibitor-added solution was shifting toward nobler direction attributed, to the transformation of nonprotective, loosely bound oxide into protective, adherent, and stable oxide film.

### 3.2. Characterization of Passive/Oxide Film

#### 3.2.1. SEM

**Figure 8:**
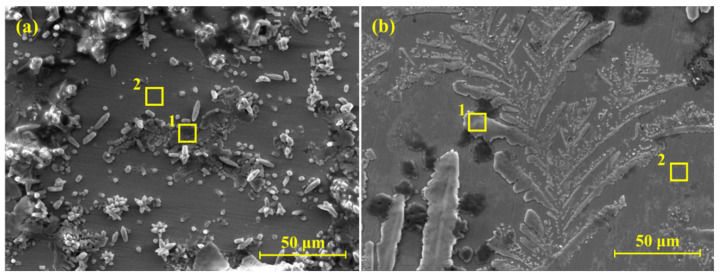
SEM images of steel rebar samples after 120 h of exposure in (**a**) bare (SCP + 3.5 wt.% NaCl) and (**b**) 2% inhibitor-added SCP + 3.5 wt.%% NaCl solution, at 1000×.

From the electrochemical studies, it was observed that the steel rebar exposed in 2% inhibitor-added solution performed best among all samples. Therefore, it was important to characterize the passive/oxide film formed on the steel rebar surface exposed in 2% inhibitor-containing solution along with bare solution for comparison after 120 h of exposure in SCP + 3.5 wt.% NaCl solution. SEM results of the rebars exposed to bare and 2% inhibitor-added solutions are shown in Figure 8a,b, respectively, at 1000×. It can be seen that the steel rebar exposed in bare solution exhibited defective and porous oxide film with crystal morphology (Figure 8a), which might be owing to the presence of NaCl coming from the solution medium. The oxide film was unevenly distributed, which resulted in a higher corrosion rate, attributed to the accumulation of NaCl compared to the steel rebar exposed in 2% inhibitor-added solution. SEM image corroborates EIS results where *R_p_* and *R_o_* were lower for the sample exposed in bare solution compared to 2% inhibitor-added solution. Agglomeration was observed at some places onto the steel rebar surface, owing to localized corrosion (Figure 8a). There was no uniform morphology in oxide/passive film, which can be seen in Figure 8a where agglomerated (mark 1 in Figure 8a) and plain (mark 2 in Figure 8a) surfaces are observed. At the plain surface, Cl^−^ ions’ concentration would be lower, while at agglomerated surface Cl^−^ ions’ would be higher. Thus, there is possibility that Cl^−^ ions can be diffused from higher (mark 1 in Figure 8a) to lower (mark 2 in Figure 8a) concentrations, which later induces the corrosion reaction, resulting in the formation of agglomerated morphology. On the other hand, the steel rebar exposed in 2% inhibitor-added solution shows dendritic growth of passive film, which can propagate at a longer duration of exposure and cover all over the surface (Figure 8b). This dendritic growth inhibited the corrosion reaction onto the steel rebar surface. Thus, highest *R_p_* and *R_o_* were observed. It was observed by Nihali et al. [47] that such type of morphology appears, owing to the phosphate layer after adding of Na_3_PO_4_ in alkaline solution. However, it is very important to know the elemental composition of oxide films. Thus, EDS was performed at plain and agglomerated surface as marks 1 and 2 in Figure 8.

The chemical compositions of oxide films are shown in Table 3. The agglomerated surface (point 1, Figure 8a) shows high amount of Cl (40.10 wt.%) and Na (55.76 wt.%). This result suggests that the agglomerated part mostly contained NaCl, which covered the surface and induced the corrosion reaction. Na content was the highest as the SCP solution containing NaOH as well as 3.5 wt.% NaCl. Ca and K were not in significant amounts and they came from SCP, where KOH and CaO were added. On the other hand, once the EDS was performed at point 2 (plain surface) in Figure 8a, Na, K, Ca, and Cl contents were found in much lower amounts. There were no N or P on the rebar surface exposed to bare solution, owing to the absence of inhibitor. It can be seen at point 2 that mostly O and Fe are observed, which reveals that it was iron oxide/hydroxide. This result suggests that Cl^−^ ions attacked on localized point onto the steel rebar surface and induced the corrosion process. Thus, lower *R_p_* and *R_o_* were observed. The EDS analysis for the sample exposed to the 2% inhibitor-containing solution at point 1 (dendrite) of Figure 8b shows the presence of 1.16 wt.% P, which confirms the formation of iron phosphate, as observed by Simesco and Idrissi [54]. The occurrence of Cl, Na, K, and Ca are owing to the composition of SCP solution and NaCl. O (2.37 wt.%) was higher compared to the sample exposed in bare solution, which revealed that 2% inhibitor may form some iron oxide/hydroxide as well as iron phosphate. The EDS was taken at point 2 (plain surface) of Figure 8b, which shows the reduced amount of P. This result suggests that the dendritic structure did not grow completely, and contained iron phosphate. Even though, at this point, P was observed, which at later exposure periods may grow and form protective oxide film. Thus, it is very important to characterize the passive film by another analytical technique. In subsequent paragraphs, the nature of passive film is discussed with the help of Raman spectroscopy and XPS.

#### 3.2.2. Raman Spectroscopy

**Figure 9:**
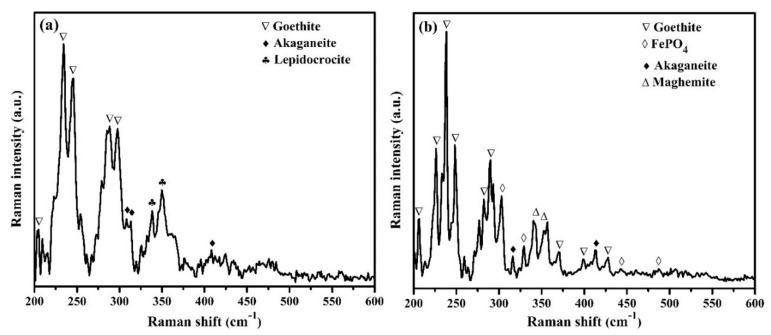
Raman spectra of passive/oxide films formed onto the rebar samples after 120 h of exposure in (**a**) bare (SCP + 3.5 wt.% NaCl) and (**b**) 2% inhibitor-added SCP + 3.5 wt.% NaCl solution.

The characterization of passive film formed on steel surface after 120 h of exposure in SCP + 3.5 wt.% NaCl (bare) and 2% inhibitor-containing solution was performed by Raman spectroscopy and results are shown in Figure 9a,b, respectively. It was found that the steel rebars exposed to the bare, as well as 2% inhibitor-containing solutions, showed α-FeOOH (goethite) and β-FeOOH (akaganeite). The attribution of the goethite in the sample exposed to the bare solution was found to be at 205, 234, 245, 288, and 297 cm^−1^ Raman shift [55,56] (Figure 9a), whereas samples in 2% inhibitor-added solution were found to be 206, 226, 238, 248, 282, 290, 370, 397, and 425 cm^−1^ [55,56] (Figure 9b). The presence of goethite corroborated the finding of Yohai et al. [31]. They found goethite in bare as well as phosphate-containing samples. The number of peaks and intensity of the samples exposed to the inhibitor-containing solution (Figure 9b) were higher compared to the samples exposed to bare solution, which suggests that goethite is more prominent in inhibitor-containing sample compared to bare, which controls the corrosion of the steel rebar exposed in SCP + 3.5 wt.% NaCl solution after 120 h of exposure. Akaganeite is a chloride-bearing iron oxide-hydroxide that is formed in a chloride-contaminated environment where Cl^–^ ions are present in its crystal structure. The akaganeite is found at 308, 314, and 410 cm^−1^ [55] in bare (Figure 9a), whereas in the presence of inhibitor, it is found at 316 and 413 cm^−1^ [55] (Figure 9b). The shifting in peak position of the samples exposed to the inhibitor-containing solutions owed to the presence of many phases. The presence of γ-FeOOH (lepidocrocite) at 338 cm^−1^ [57] and 349 cm^−1^ [55] in the sample exposed to the bare solution (Figure 9a) was owing to the initiation of corrosion reaction in SCP + 3.5 wt.% NaCl solution. However, the sample in 2% inhibitor-containing solution showed other extra phases, which were γ-Fe_2_O_3_ (maghemite) at 343 cm^−1^ and 352 cm^−1^ [55] (Figure 9b) as well as FePO_4_ at 303, 330, 442, and 487 cm^−1^ [58,59] (Figure 9b). The formation of maghemite and FePO_4_ will be described in Discussion section. However, maghemite and FePO_4_ are thermodynamically very stable and sparingly soluble. Thus, the steel sample exposed to the 2% inhibitor-containing solution showed highest *R_p_* and *R_o_* and lowest corrosion compared to other samples.

#### 3.2.3. XPS Analysis

**Figure 10:**
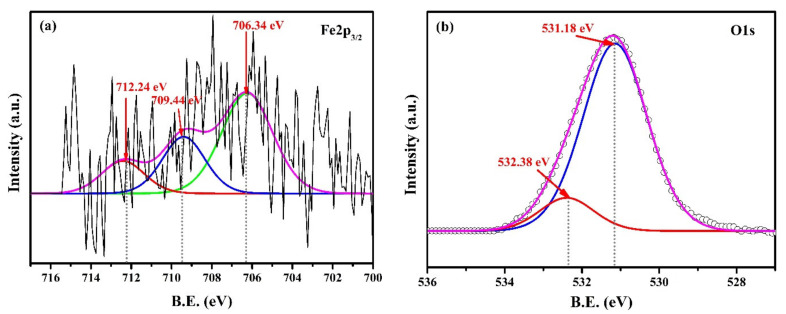
XPS spectra of oxide film (**a**) Fe2p_3/2_ and (**b**) O1s formed on steel rebar surface after 120 h of exposure in SCP + 3.5 wt.% NaCl solution.

**Figure 11:**
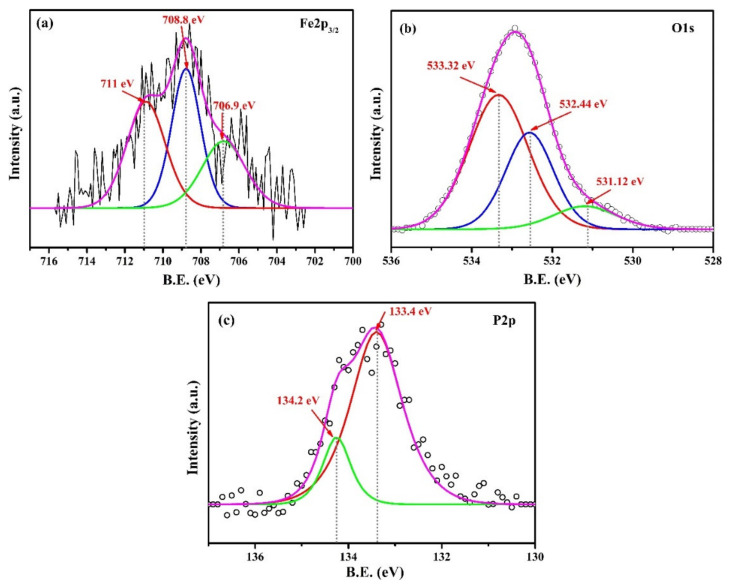
XPS spectra of oxide film (**a**) Fe2p_3/2_, (**b**) O1s, and (**c**) P2p formed on steel rebar surface after 120 h of exposure in 2% inhibitor-added SCP + 3.5 wt.% NaCl solution.

The XPS of passive/oxide film formed on the surface of steel rebars exposed to the bare and 2% inhibitor-containing solution after 120 h of exposure are shown in Figure 10 and Figure 11 to confirm the observations of Raman spectroscopy, respectively. The spectrum of Fe2p_3/2_ in bare sample is noisy and poorly defined (Figure 10a), owing to the formation of unstable and porous thick corrosion products, i.e., Fe(OH)_2_, which hinder getting a clear spectrum [31,60]. There are three Fe2p_3/2_ peaks fitted at 706.34 eV, 709.44 eV [61,62], and 712.24 eV [63,64] for metallic iron (Fe), Fe(II), and Fe(III) [61,65] in Figure 10a, respectively. From this figure, it can be seen that broadening in peaks of metallic Fe and Fe(II), i.e., Fe(0)/Fe(II) was higher, owing to the bare surface and formation of porous Fe(OH)_2_, respectively [66], which influenced the corrosion reaction in chloride-contaminated SCP solution. However, Fe(OH)_2_ was not found in Raman spectroscopy due to its instability or transformation into other phases. There is another possibility that Fe(OH)_2_ is very thin and cannot be detected by Raman spectroscopy, owing to the limitation of this instrument. However, XPS can detect up to 10 nm thickness of oxide or film [61]. Thus, it was observed by XPS. This result corroborated SEM (Figure 8a), where plain surface was observed. The plain surface was owing to the metallic Fe and initial corrosion reaction, which formed Fe(OH)_2_. It is reported that, if Fe(II)/Fe(III) content is higher (as broadening observed in Figure 10a for Fe(II)), then more oxygen vacancies are formed in passive film [67]. The binding-energy value of Fe(II) at 709.44 eV was attributed to the FeO/Fe(OH)_2_ [60], which is very unstable and transforms into Fe(III). The presence of Fe(III) at 712.24 eV in the sample exposed to the bare solution might have been owing to the formation of FeOOH, i.e., lepidocrocite, akageneite, and goethite [64], as also evidenced in Raman spectroscopy (Figure 9a).

The fitting of two peaks in O1s at 531.18 eV and 532.38 eV were attributed to the O^2−^ and OH^−^, respectively. There was shifting in 1 eV toward higher binding energy in O1s compared to reported values [31], owing to the formation of different oxides/hydroxides such as goethite, lepidocrocite, and akageneite, which cause interference and shifting of binding energy.

XPS results of the steel rebar exposed in 2% inhibitor-containing solution are shown in Figure 11. The Fe2p_3/2_ peak was deconvoluted into three peaks at 706.9 eV [61,62], 708.8 eV, and 711 eV for metallic iron (Fe), Fe(II), and Fe(III), respectively [31] in Figure 11a. Metallic Fe and Fe(II) at 706.9 eV and 708.8 eV were attributed to the plain surface, as observed in Figure 8b, and initiation of corrosion phenomena where Fe(OH)_2_ formed in SCP + 3.5 wt.% NaCl solution. It was observed that once the 2% inhibitor was added in solution, the Fe(III)/Fe(II) content in passive film increased (broadening in peaks) compared to Fe(II) (Figure 11a), which induced forming protective passive film in SCP + 3.5 wt.% NaCl solution [33,67]. The addition of 2% inhibitor increased the thickness of passive film in steel rebar surface. Therefore, the metallic Fe content decreased (Figure 11a). The broadening in Fe(III) was attributed to the involvement of different protective iron oxide/hydroxide such as goethite, maghemite, and iron phosphate. The binding energy value of Fe2p_3/2_ at 711 eV was attributed to the presence of γ-Fe_2_O_3_ (maghemite), as reported earlier [68,69,70]. The detection of maghemite by XPS correlated with the results of Raman spectroscopy (Figure 9b).

O1s was fitted with three peaks at 531.12 eV, 532.44 eV, and 533.32 eV, attributed to the O^2−^, OH^−^, and PO_4_^3−^ ions (Figure 11b), respectively. The shifting in higher binding energy of OH^−^ and PO_4_^3−^ was attributed to the presence of goethite and iron phosphate, respectively. Phosphate ions react with Fe and form iron phosphates. Therefore, shifting in binding energy of O1s was observed.

The XPS peak that appeared for phosphorous was deconvoluted (Figure 11c) into two peaks at 133.4 eV and 134.2 eV for P2p_3/2_ and P2p_1/2_, respectively. Presence of P2p revealed the presence of phosphate and it was due to the formation of FePO_4,_ as observed earlier [71]. The formation of FePO_4_ on the sample surface was also observed by Raman spectroscopy (Figure 9b), which corroborated the XPS results.

## 4. Discussion

During the initial period of exposure, i.e., after 1 h, there would be interaction of solution and Cl^−^ ion with iron (Fe) to initiate the corrosion process by following reactions [7,72]:

(5) $$\mathrm{Fe}\to {\mathrm{Fe}}^{2+}+2e^{-}\;(\mathrm{anodic})$$

(6) $$\frac{1}{2}O_{2}+H_{2}O+2e^{-}\to 2OH^{-}\;(\mathrm{cathodic})$$

Overall reaction may be written as:

(7) $$\mathrm{Fe}+\frac{1}{2}O_{2}+H_{2}O\to \mathrm{Fe}{\left(OH\right)}_{2}$$

However, it is well known that Fe(OH)_2_ is very unstable, owing to the Fe^2+^ oxidation state as well as being amphoteric in nature. It was detected by XPS (Figure 10 and Figure 11) and again reacted with Cl^−^ ions in solution (SCP + 3.5 wt.%NaCl) and formed acidic ferrous chloride (Equation (9)) [73]:

(8) $$\mathrm{Fe}{\left(OH\right)}_{2}+2Cl^{-}\to \mathrm{Fe}OCl_{2}^{--}+H_{2}O$$

(9) $$\mathrm{Fe}OCl_{2}^{--}+H_{2}O\to \mathrm{Fe}Cl_{2}+2OH^{-}$$

Again, FeCl_2_ reacted with H_2_O and forms Fe(OH)_2_ as well as HCl (Equation (10)). Due to the release of HCl, the solution became acidic and induced the anodic dissolution of steel rebar [73].

(10) $$\mathrm{Fe}Cl_{2}+2H_{2}O\to \mathrm{Fe}{\left(OH\right)}_{2}+2H\mathrm{Cl}$$

Ultimately, Fe(OH)_2_ would form during initiation of corrosion (Equations (7) and (10)) and, in the meantime, oxygen diffuses at steel/solution interface and transforms the Fe(OH)_2_ into FeO.OH.

(11) $$2\mathrm{Fe}{\left(OH\right)}_{2}+\frac{1}{2}O_{2}\to 2\mathrm{Fe}O.OH+H_{2}O$$

The above corrosion reactions occur on steel rebar surface exposed in bare as well as inhibitor-containing SCP + 3.5 wt.% NaCl solutions. It was reported by other researchers that FeO.OH is α-FeOOH (goethite), which controls the corrosion of steel rebar [74,75]. Keiser et al. found that FeO.OH is very adherent and stable to control the deterioration of steel rebar than other iron oxides [76]. However, in the case of inhibitor, FeO.OH transforms into Fe_2_O_3_ (Equation (12)), which is maghemite (γ-Fe_2_O_3_), as observed by Raman spectroscopy (Figure 9b) and XPS (Figure 11a).

(12) $$2\mathrm{Fe}O.OH\to Fe_{2}O_{3}+H_{2}O$$

In the meantime, hydrolysis of APMB (NH_4_H_2_PO_4_) inhibitor may occur, which locally reduces the pH of solution, owing to the formation of H_3_PO_4_ (Equation (13)) during electrochemical reaction at steel rebar/solution interface [40]. However, initially it was observed that once the inhibitor was added, the pH of SCP + 3.5 wt.% NaCl solution increased due to the liberation of NH_4_OH (Equation (13)). But once the exposure periods were increased, the pH of the solution reduced because of the formation of H_3_PO_4_ inside the solution.

(13) $$NH_{4}H_{2}PO_{4}+H_{2}O\to H_{3}PO_{4}+NH_{4}OH$$

H_3_PO_4_ reduced the pH, owing to the strong acidic nature, resulting in lower total impedance (Figure 4b), which was observed for higher amounts of inhibitors i.e., 3% inhibitor. Thus, at longer duration of exposure, *R_p_* and *R_o_* decreased (Figure 6, Table 1). Moreover, for the sample in 2% inhibitor-added solution, the *R_p_* and *R_o_* initially decreased up to 24 h but once the proper reaction occurred, their values increased up to 120 h (Figure 6, Table 1). The highest *R_p_* and *R_o_* of steel rebar exposed in 2% APMB inhibitor-added solution might have been owing to the reaction of Fe(OH)_2_ (Equations (7) and (10)) with APMB [77] where a proper amount of H_3_PO_4_ formed. H_3_PO_4_ led to forming the iron phosphate (Equations (14)–(16)), whereas in 1% inhibitor, there was no significant amount of phosphate ions to react with iron (Fe) and form iron phosphate (FePO_4_). However, in the case of 3% inhibitor, a significant amount of phosphoric acid was released, which locally reduced the pH of solution, started to dissolve the Fe(OH)_2_, and induced the corrosion reaction at steel rebar/solution interface. After 120 h of exposure, pH was measured for 1, 2, and 3% inhibitor-added solutions and these were found to be reduced from 12.65, 12.67, and 12.68 (measured during preparation of solution) to 12.42, 12.50, and 12.08 at 20 °C, respectively. However, bare solution maintained its pH, i.e., 12.60, even after 120 h of exposure of steel rebar. This result suggests that there was no occurrence of atmospheric carbonation rather than the effect of inhibitor in reduction of pH. This result suggests that once the amount of inhibitor increased, the pH of resulting solution became acidic, owing to the acidification by H_3_PO_4_ (Equation (13)). Therefore, it was concluded that 2% inhibitor is the optimal amount of 0.5 M APMB to form the protective oxide/passive film, i.e., FePO_4_, which was observed by Raman spectroscopy (Figure 9b) and XPS (Figure 11b).

(14) $$\begin{array}{c} \mathrm{Fe}{\left(OH\right)}_{2}+2H_{3}PO_{4}\to \mathrm{Fe}{\left(H_{2}PO_{4}\right)}_{2}+2H_{2}O\\ ↓\\ \left(\mathrm{primary}\mathrm{iron}\mathrm{phosphate}\right) \end{array}$$

(15) $$\begin{array}{c} \mathrm{Fe}{\left(H_{2}PO_{4}\right)}_{2}\to \mathrm{Fe}HPO_{4}+H_{3}PO_{4}\\ ↓\\ \left(\mathrm{secondary}\mathrm{iron}\mathrm{phosphate}\right) \end{array}$$

(16) $$\begin{array}{c} 4\mathrm{Fe}HPO_{4}+O_{2}\to 4\mathrm{Fe}PO_{4}+2H_{2}O\\ \;\;\;\;\;\;\;\;\;\;\;\;\;\;\;\;\;\;\;\;\;↓\\ \;\;\;\;\;\;\;\;\;\;\;\;\;\;\;\;\;\;\;\;\;(\mathrm{tertiary}\mathrm{iron}\mathrm{phosphate}) \end{array}$$

Fe(OH)_2_ reacted with H_3_PO_4_ released from APMB inhibitor (Equation (13)) during exposure in solution and formed primary iron phosphate, i.e., Fe(H_2_PO_4_)_2_ (Equation (14)), and secondary iron phosphate, i.e., FeHPO_4_ (Equation (15)). These phosphates are very unstable and soluble in alkaline environment, which might have been transformed into tertiary iron phosphate, i.e., FePO_4_ (Equation (16)), by reacting with oxygen. FePO_4_ is very stable and insoluble in alkaline as well as slightly acidic conditions [78]. Thus, APMB inhibitor-containing steel rebar exhibited higher impedance, which reduced the corrosion reaction. Therefore, it can be said that 2% inhibitor is the optimal amount to form proper passive film onto the steel rebar surface.

## 5. Conclusions

From the above results and discussion, the following conclusions can be drawn:

OCP of steel rebar exposed in inhibitor-containing solution is nobler compared to bare (without inhibitor) solution but gradually shifted toward active direction with exposure period, owing to the acidification of the solution. However, OCP of the rebar sample exposed to 2% inhibitor-added solution shifted toward active direction up to 24 h of exposure thereafter. Ennobling in OCP was observed owing to the formation of protective passive film.

(1) EIS results corroborated with the OCP where initially up to 24 h of exposure, *R_p_* and *R_o_* gradually decreased but, once the exposure period reache up to 120 h, their values increased, which revealed the formation of passive film onto the steel rebar in 2% inhibitor-containing solution.
(2) The steel rebar exposed in 2% inhibitor-added SCP + 3.5 wt.% NaCl solution showed 90% inhibition efficiency after 1 h of exposure but its value decreased to 62.48% once the exposure periods were extended up to 120 h.
(3) Potentiodynamic studies revealed that the steel rebar exposed to 2% inhibitor-added solution exhibited lower in cathodic and anodic current density compared to the rebar exposed to bare solution after 120 h of exposure.
(4) SEM results of the steel rebar exposed to 2% inhibitor-containing solution show dendritic growth of the oxides on the surface after 120 h of exposure while the steel rebar exposed to the bare solution exhibited agglomeration of oxide products on the surface.
(5) Raman spectroscopy and XPS confirmed the formation of thermodynamically stable and sparingly soluble goethite, maghemite, and iron phosphate (FePO_4_) as passive/oxide film onto the surface of steel rebar exposed to 2% inhibitor-containing solution. Thus, corrosion rate reduced at longer duration of exposure in SCP + 3.5 wt.% NaCl solution.

## Figures and Tables

**Table 1:** Details of the solutions used for experiments.

Serial No.	Amount of APMB Inhibitor (v/v%)	pH	Solution
1.	0.00	12.65	SCP + 3.5 wt% NaCl
2.	1.00	12.65	SCP + 3.5 wt% NaCl + 1% APMB
3.	2.00	12.67	SCP + 3.5 wt% NaCl + 2% APMB
4.	3.00	12.68	SCP + 3.5 wt% NaCl + 3% APMB

**Table 2:** Electrochemical parameters of samples exposed in SCP + 3.5 wt.% NaCl solution with and without inhibitor.

Time (h)	Sample ID	Electrochemical Parameters						Efficiency (%)
		*R_s_* (Ω·cm^2^)	*CPE1*		*R_o_* (kΩ·cm^2^)	*CPE2*		
			*Q_1_* (1 × 10^−5^) (Ω^−1^·cm^−2^·s*^n^*)	*n_1_*		*Q_2_* (1 × 10^−5^) (Ω^−1^·cm^−2^·s*^n^*)	*n_2_*	
1	Bare	9.96	21.0	0.78	0.87	232.8	0.70	-
	1%	9.80	14.2	0.82	4.61	54.4	0.74	81.13
	2%	10.09	10.2	0.85	8.71	24.5	0.80	90.01
	3%	11.78	13.1	0.82	5.24	50.7	0.75	83.40
24	Bare	11.48	16.6	0.80	2.16	182.4	0.73	-
	1%	9.32	15.7	0.81	4.47	55.0	0.74	51.68
	2%	11.64	12.3	0.83	6.45	42.4	0.78	66.51
	3%	12.89	13.2	0.82	4.70	52.0	0.74	54.04
120	Bare	11.12	23.8	0.76	2.66	155.7	0.73	-
	1%	10.54	22.0	0.77	2.26	175.4	0.68	−17.70
	2%	12.59	11.7	0.83	7.09	34.8	0.79	62.48
	**3%**	12.60	20.6	0.77	4.04	52.7	0.73	34.16

**Table 3:** EDS analysis of samples in SCP + 3.5 wt.% NaCl solution after 120 h of exposure.

Solution	Figure	Point	Element (wt.%)							
			O	N	P	Cl	Na	K	Ca	Fe
Bare	8a	1	0.54 (±0.02)	-	-	40.1 (±2.40)	55.76 (±1.95)	1.34 (±0.08)	0.29 (±0.02)	1.97 (±0.09)
		2	2.1 (±0.05)	-	-	0.18 (±0.01)	0.35 (±0.02)	0.24 (±0.02)	0.23 (±0.02)	96.90 (±7.27)
2% inhibitor	8b	1	2.37 (±0.10)	0	1.16 (±0.080)	2.87 (±0.15)	3.21 (±0.14)	2.6 (±0.16)	1.37 (±0.05)	86.42 (±5.27)
		2	1.73 (±0.04)	1.1 (±0.04)	0.16 (±0.01)	0.23 (±0.01)	0.48 (±0.013)	0.22 (±0.01)	0.2 (±0.01)	95.88 (±4.99)
